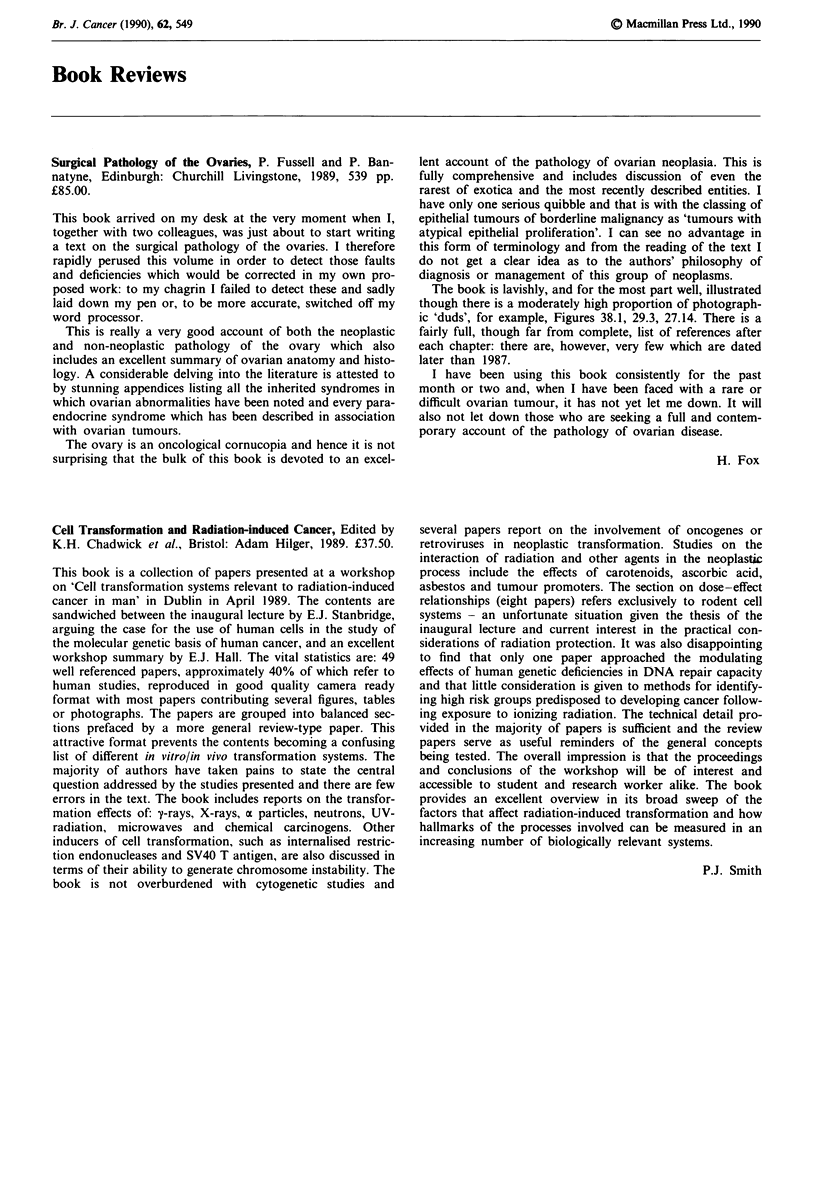# Cell Transformation and Radiation-induced Cancer

**Published:** 1990-09

**Authors:** P.J. Smith


**Cell Transformation and Radiation-induced Cancer,** Edited by K.H. Chadwick *et al.*, Bristol: Adam Hilger, 1989. £37.50.

This book is a collection of papers presented at a workshop on 'Cell transformation systems relevant to radiation-induced cancer in man' in Dublin in April 1989. The contents are sandwiched between the inaugural lecture by E.J. Stanbridge, arguing the case for the use of human cells in the study of the molecular genetic basis of human cancer, and an excellent workshop summary by E.J. Hall. The vital statistics are: 49 well referenced papers, approximately 40% of which refer to human studies, reproduced in good quality camera ready format with most papers contributing several figures, tables or photographs. The papers are grouped into balanced sections prefaced by a more general review-type paper. This attractive format prevents the contents becoming a confusing list of different *in vitro/in vivo* transformation systems. The majority of authors have taken pains to state the central question addressed by the studies presented and there are few errors in the text. The book includes reports on the transformation effects of: γ-rays, X-rays, α particles, neutrons, UV-radiation, microwaves and chemical carcinogens. Other inducers of cell transformation, such as internalised restriction endonucleases and SV40 T antigen, are also discussed in terms of their ability to generate chromosome instability. The book is not overburdened with cytogenetic studies and several papers report on the involvement of oncogenes or retroviruses in neoplastic transformation. Studies on the interaction of radiation and other agents in the neoplastic process include the effects of carotenoids, ascorbic acid, asbestos and tumour promoters. The section on dose–effect relationships (eight papers) refers exclusively to rodent cell systems – an unfortunate situation given the thesis of the inaugural lecture and current interest in the practical considerations of radiation protection. It was also disappointing to find that only one paper approached the modulating effects of human genetic deficiencies in DNA repair capacity and that little consideration is given to methods for identifying high risk groups predisposed to developing cancer following exposure to ionizing radiation. The technical detail provided in the majority of papers is sufficient and the review papers serve as useful reminders of the general concepts being tested. The overall impression is that the proceedings and conclusions of the workshop will be of interest and accessible to student and research worker alike. The book provides an excellent overview in its broad sweep of the factors that affect radiation-induced transformation and how hallmarks of the processes involved can be measured in an increasing number of biologically relevant systems.

P.J. Smith